# Quantitative and individualized assessment of the learning curve in preoperative planning of the acetabular cup size in primary total hip arthroplasty

**DOI:** 10.1007/s00402-021-03848-6

**Published:** 2021-03-11

**Authors:** W. Waldstein, P. A. Bouché, C. Pottmann, M. Faschingbauer, P. R. Aldinger, R. Windhager, C. Merle

**Affiliations:** 1 Department of Orthopedic and Trauma Surgery, Vienna General Hospital, Medical University of Vienna, Waehringer Guertel 18-20, 1090 Vienna, Austria; 2 Department of Orthopaedic Surgery, Hôpital Cochin, Assistance Publique-Hôpitaux de Paris, Université Paris Descartes, Paris, France; 3 Department of Orthopedic Surgery, University of Ulm, Ulm, Germany; 4 Department of Orthopaedic Surgery Paulinenhilfe, Diakonie Klinikum, Stuttgart, Germany; 5 Department of Orthopaedics and Trauma Surgery, Heidelberg University Hospital, Schlierbacher Landstrasse 200a, 69118 Heidelberg, Germany

**Keywords:** Learning curve, Planning quality, Templating, Total hip arthroplasty, Acetabular cup, Process quality

## Abstract

**Introduction:**

The aim of the present study was to investigate the learning curves of 2 trainees with different experience levels to reach proficiency in preoperative planning of the cup size based on learning curve cumulative summation (LC-CUSUM) statistics and a cumulative summation (CUSUM) test.

**Materials and methods:**

One-hundred-twenty patients who had undergone primary total hip arthroplasty with a cementless cup were selected. Preoperative planning was performed by an experienced orthopedic surgeon. Trainee 1 (student) and trainee 2 (resident) planned the cup size. The trainees were blinded to the preoperative plan and the definitive cup size. Only after a cup size was chosen, the trainees were unblinded to the preoperative plan of the surgeon. LC-CUSUM was applied to both trainees to determine when proficiency in determining the appropriate cup size was reached. A CUSUM test was applied to ensure retention of proficiency.

**Results:**

With reference to the preoperative plan of the surgeon, LC-CUSUM indicated proficiency after 94 planning attempts for trainee 1 and proficiency after 66 attempts for trainee 2, respectively. Trainee 1 and 2 maintained proficiency thereafter. With reference to the definitive cup size, LC-CUSUM did not signal competency within the first 120 planning attempts for trainee 1. Trainee 2 was declared competent after 103 attempts and retained competency thereafter.

**Conclusions:**

LC-CUSUM/CUSUM allow for an individualized, quantitative and continuous assessment of planning quality. Based on LC-CUSUM statistics, the two trainees of this study gain proficiency in planning of the acetabular cup size after 50–100 attempts when an immediate feedback is provided. Previous experience positively influences the performance. The study serves as basis for the medical education of students and residents in joint replacement procedures**.**

## Introduction

Contemporary total hip arthroplasty (THA) aims for an accurate restoration of physiological joint biomechanics [17]. Anteroposterior (AP) pelvis radiographs are frequently used in preoperative templating for THA because of additional information regarding the anatomy of the pelvis and the contralateral hip, and because they allow for an evaluation of leg length discrepancies [11, 14].

In preoperative planning, accurate and reliable assessment of the appropriate acetabular component size is important. Occult periprosthetic acetabular fractures after press-fit implantation of the cup during primary THA were observed on postoperative CT scans in 8% of cases [9]. Only the usage of self-locking cups was identified as relevant risk factor; however, gender did not influence the rate of periprosthetic fractures [9]. Sharkey et al. suggested that oversizing of the cup may be associated with an increased rate of periprosthetic acetabular fractures [22] whereas undersizing may be associated with early loosening as a result of insufficient press-fit [18]. Additional benefits of accurate preoperative planning include shortened surgical time, and reduced complications with equipment and an improved reconstruction of individual joint geometry [11]. According to the literature, preoperative planning of the acetabular cup size has a high accuracy and predicts the definite implant within ± one size in 80% to 100% [8, 11, 21, 24; 26].

Statistical process control charts have been adapted to quantitatively assess attainment and maintenance of proficiency in medicine [6, 7, 20]. The cumulative summation test (CUSUM) assumes that a process remains within an acceptable limit and that it is ‘in control’ (null hypothesis, H_0_) [19]. As soon as a process is deviating from a target of adequate performance (process is out of control) the null hypothesis is rejected and the alternative hypothesis (H_1_) is accepted [4]. The limitation of the CUSUM test is that an initial learning process is often associated with poor performance triggering unacceptable failure rates [4]. Therefore, the cumulative summation test for learning curve (LC-CUSUM) was introduced by Biau and Porcher in 2008 [2, 3]. Bases on predefined acceptable and unacceptable failure rates, LC-CUSUM assumes that a process is initially out of control and indicates when a process is in control.

No study has evaluated the learning process associated with the attainment of proficiency in accurate preoperative planning of the acetabular cup size in primary cementless THA.

The aim of the present study was to investigate the learning curves of 2 trainees with different experience levels to reach proficiency in preoperative planning of the acetabular cup size using LC-CUSUM, and to monitor the quality of attained proficiency using the CUSUM test.

## Materials and methods

In the current retrospective study, 120 patients with primary end-stage hip osteoarthritis were randomly selected out of a consecutive series of 597 patients who had undergone primary THA with a cementless hemispherical press-fit cup (Protocyl cup, MicroPort Orthopedics, Inc, Arlington, TN, USA or DSP cup, OS Orthopedic Services, Mainhausen, Germany) and a cementless custom-made stem [1] for primary osteoarthritis (OA) of the hip between June 2009 and December 2009. The Protocyl cup was utilized in 93% and the DSP cup in 7% of cases, respectively. Calibrated preoperative AP radiograph of the pelvis were available. In each case, a standardized protocol was used to achieve reproducible projections. Low-centered AP radiographs of the pelvis were taken in the supine position with both legs 15° internally rotated using a leg retainer and the crosshair of the beam centered on the symphysis pubis. In order to correct for the effects of magnification, a metal calibration sphere of 25 mm was placed on the inner thigh at the level of the femoral head in AP projection. A previous study on the same dataset demonstrated that radiographs were precisely calibrated with reference to CT scans [XY].

All images were retrieved in DICOM format from a picture archiving and communication system (PACS). The study was conducted in accordance with the Declaration of Helsinki of 1975 revised in 2013, and the protocol was approved by the local Ethics Committee (S-272/2009). The study included 68 female (57%) and 52 male patients (43%) with a mean age of 60 years (SD 7.5, range 42–79) and a mean body mass index (BMI) of 26.9 kg/m2 (SD 4.4). Primary THA was performed on 62 left (52%) and 58 right (48%) hip joints.

For all patients, preoperative AP pelvis radiographs were obtained in supine position according to a standardized protocol. To correct for effects of magnification, a 25 mm metal calibration sphere was positioned on the inner thigh at the height of the femoral head. The crosshair of the beam was centered on the pubic symphysis and both legs were internally rotated by 15 degrees using a foot retainer. The tube-to-film distance was 1150 mm, with the tube orientation perpendicular to the table.

**Fig. 1:**
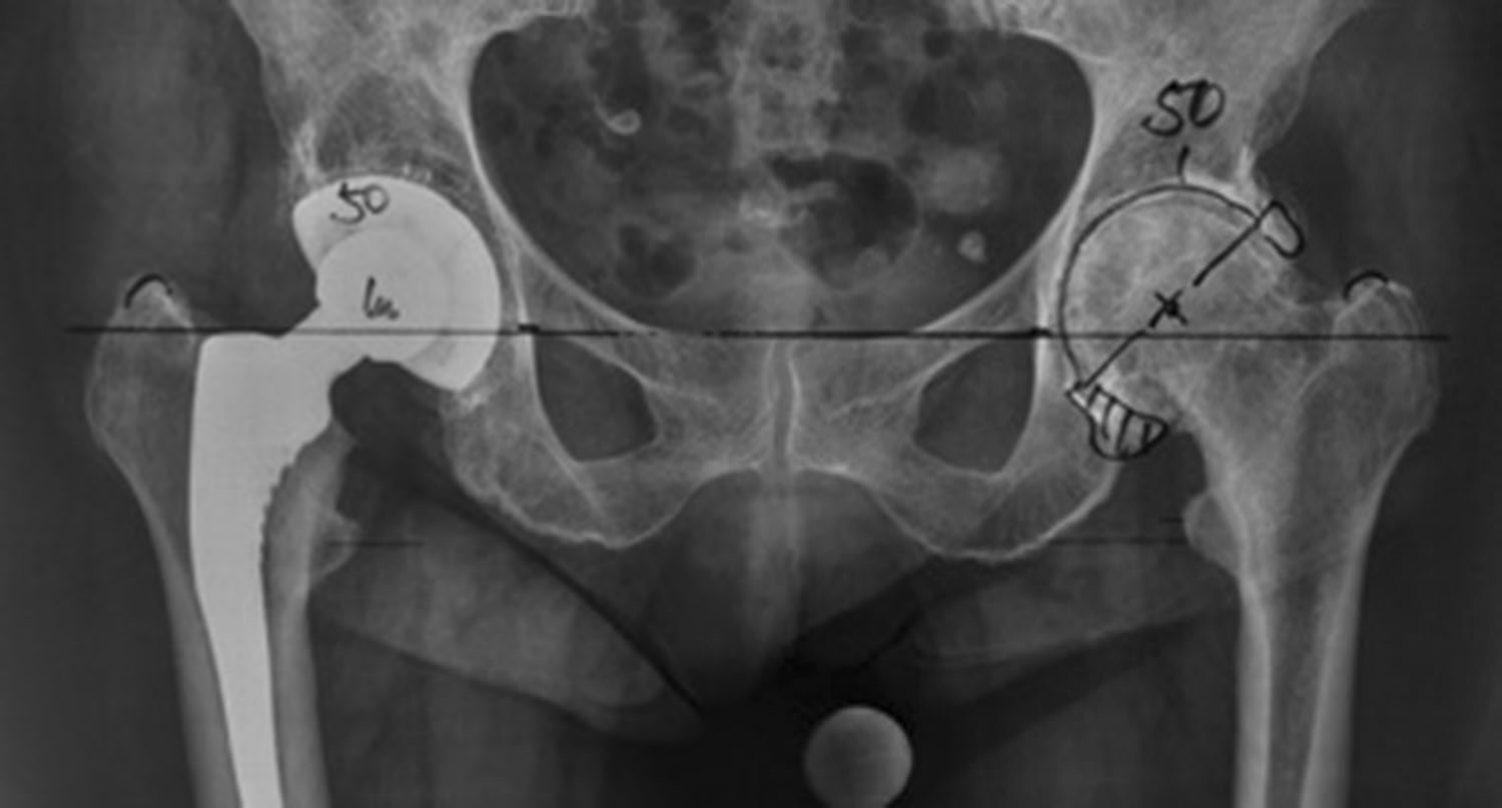
Preoperative templating of a 50-mm hemispherical press-fit cup in a 79-year old female patient by the senior surgeon

The templated cup size and final implant size were retrieved in all patients. Preoperative planning was manually performed on AP pelvis radiographic films by a senior orthopedic surgeon with more than 30 years of experience in hip replacement who subsequently performed all surgeries. Intraoperative positioning and sizing of the cup followed the concept of a ‘conventional reaming technique’ where reaming in the transverse plane extended to the lateral lamina (‘true floor’) of the acetabular fossa. The preoperative planning aimed to position the cup with medialization to the true floor with an inclination 40 ± 10° and an antomic reconstruction of the height of the center of rotation (COR). All analog radiographs with the respective preoperative plan directly marked on the film were subsequently digitalized (Fig. 1).

Trainee 1 and 2 were blinded to the preoperative plan of the surgeon and the size of the definitive implant. Trainee 1 was a 6th year medical student with no previous experience in templating for THA. Trainee 1 received a structured introduction which included an analysis of the quality of radiographs, the identification of relevant anatomic landmarks, and the measurement of acetabular offset (AO) [15], femoral offset (FO) [16] and the center of rotation (COR), respectively. In cases of advanced OA, the contralateral side was taken into consideration to restore the COR. Using a validated software (TraumaCad, Voyant Health, Petach-Tikva, Israel) [23] with the appropriate templates, the following steps were performed in all patients: (1) calibration of the AP pelvis radiograph by means of the 25 mm marker, (2) drawing of the trans-teardrop line as horizontal reference between the distal aspects of the teardrops, and (3) the identification of relevant anatomic acetabular landmarks (base of teardrop, ilio-ischial line and superolateral margin of the acetabulum. The preoperative planning aimed to position the cup with medialization to the true floor with an inclination 40 ± 10° and an anatomic reconstruction of the height of the center of rotation (COR). The positioning and sizing of the cup followed the concept of a ‘conventional’ reaming technique where the inferomedial margin is leveled with the teardrop, the medial border approximates the lateral wall of the teardrop and superior margin has a full bony coverage at an inclination angle of 40 ± 10° [13]. After the cup size was determined, trainee 1 was unblinded to the preoperative plan of the surgeon. Templating of more than ± one size was considered as failure.

Trainee 2 was an orthopedic resident in the final year of residency with previous experience in templating approximately 200 THAs. Following the same steps and templating rationale, the preoperative cup size determined. After the appropriate size was identified, trainee 2 was unblinded to the preoperative plan of the surgeon.

The postoperative radiographs were not available for this study. The absence of these radiographs was used to assess the influence of a missing feedback mechanism on the learning process.

Statistical Analyses:

To determine when proficiency was reached, LC-CUSUM was applied to both trainees. The LC-CUSUM test presumes that the trainee is not proficient at the start of monitoring and signals when the trainee has reached the acceptable predefined level of performance. Therefore, the null hypothesis (H0) is that the process is out of control (trainee’s performance deviates from adequate performance, p0, by at least delta), and an alternative hypothesis (H1) is that process is in control (trainee’s performance is equal to the adequate performance). The trainee is considered not proficient as long as the LC-CUSUM score remains below the limit h. When the LC-CUSUM score crosses this limit, the trainee is considered to be proficient. We considered p0 = 15% as the criteria for an acceptable failure rate, p1 = 30% as an unacceptable failure rate, and delta = 0.075 as the acceptable deviation from adequate performance. Based on these settings and using computer simulation (10,000 simulations of series of 120 procedures) a limit of *h* = 1.63 was chosen for LC-CUSUM test to provide the following alarm discovery rates over 120 procedures: true discovery rate (TDR; here the probability of an alarm being raised over 100 procedures if the true performance of the surgeon is 15% failure, i.e. the student is proficient) of 94% and false discovery rate (FDR; here the probability of an alarm being raised over 120 procedures if the true performance of the surgeon is 30% failure, i.e. the surgeon is not proficient) of 10%. A standard CUSUM test was applied to both trainees once they demonstrated competency to ensure retention of proficiency. A limit *h* = 4.295 was chosen for the CUSUM test to provide a TDR (here the probability of an alarm being raised over 120 procedures if the true performance of the trainee is 30% failure) of 96% and a false discovery rate (here the probability of an alarm being raised over 120 procedures if the true performance of the trainee is 15% failure) of 5.79%.

## Results

The precision of trainee 1 and trainee 2 to predict the planned cup size and the size of the definite implant is shown in Table 1. With reference to the preoperative plan of the surgeon, the failure rate (± one size) was higher for women (trainee 1: 29%, trainee 2: 19%) compared to men (trainee 1: 13%, trainee 2: 10%). With reference to the definitive cup size, similar failure rates were observed for women (trainee 1: 23%, trainee 2: 19%) and men (trainee 1: 24%, trainee 2: 16%). The overall precision of the surgeon to predict the definitely used cup size was 94% (*n* = 113). In 5 cases (4%) the surgeon planned the cup size two sizes smaller compared to the intraoperatively used cup size. In two of these 5 cases, the preoperative plan of trainee 1 and trainee 2 was also two sizes smaller than the definite cup size.

**Table 1:** The precision of trainee 1 and trainee 2 to predict the planned cup size and the definitely used cup size is shown in total numbers and percentages

	Planned cup size (*n* = 120)				Used cup size (*n* = 120)			
	Exactly as planned	+ one size	− one size	Total rate	Exactly as used	+ one size	− one size	Total rate
Surgeon	N/A	N/A	N/A	N/A	57 (48%)	18	38	94%
Trainee 1 (n)	39 (33%)	28	29	80%	41 (34%)	25	26	77%
Trainee 2 (n)	53 (44%)	24	26	86%	46 (38%)	27	26	83%

Trainee 1: 6th year medical student; Trainee 2: 6th year orthopedic resident

In 56 out 68 female patients (82%), a cup diameter between 46 and 52 mm was implanted. The most frequently planned cup had a 50 mm diameter in females (*n* = 22; 32%). In 45 out of 52 male patients (87%), a cup diameter between 52 and 58 mm was implanted. A 54 mm cup was most frequently utilized in males (n = 14; 27%).

### CUSUM graph

**Fig. 2:**
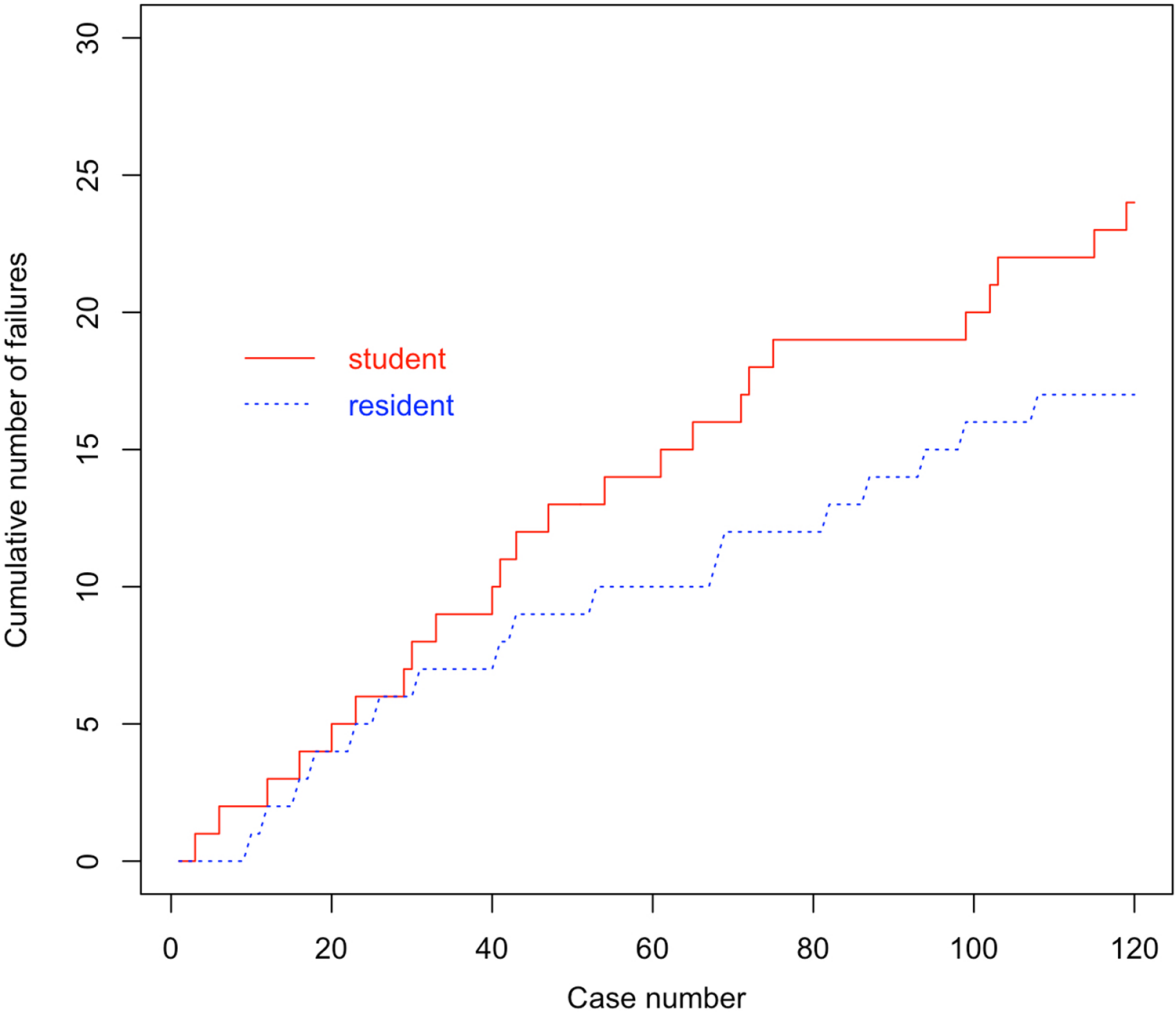
The cumulative number of failures of trainee 1 (student) and trainee 2 (resident) with reference to the cup size template

With reference to preoperative templating, the trainee 1 had 13 outliers in his early 50 planning attempts, and 7 outliers in his late 50 planning attempts, respectively. Trainee 2 had 9 outliers in his early 50 attempts, and 7 outliers in his late 50 attempts, respectively (Fig. 2).

**Fig. 3:**
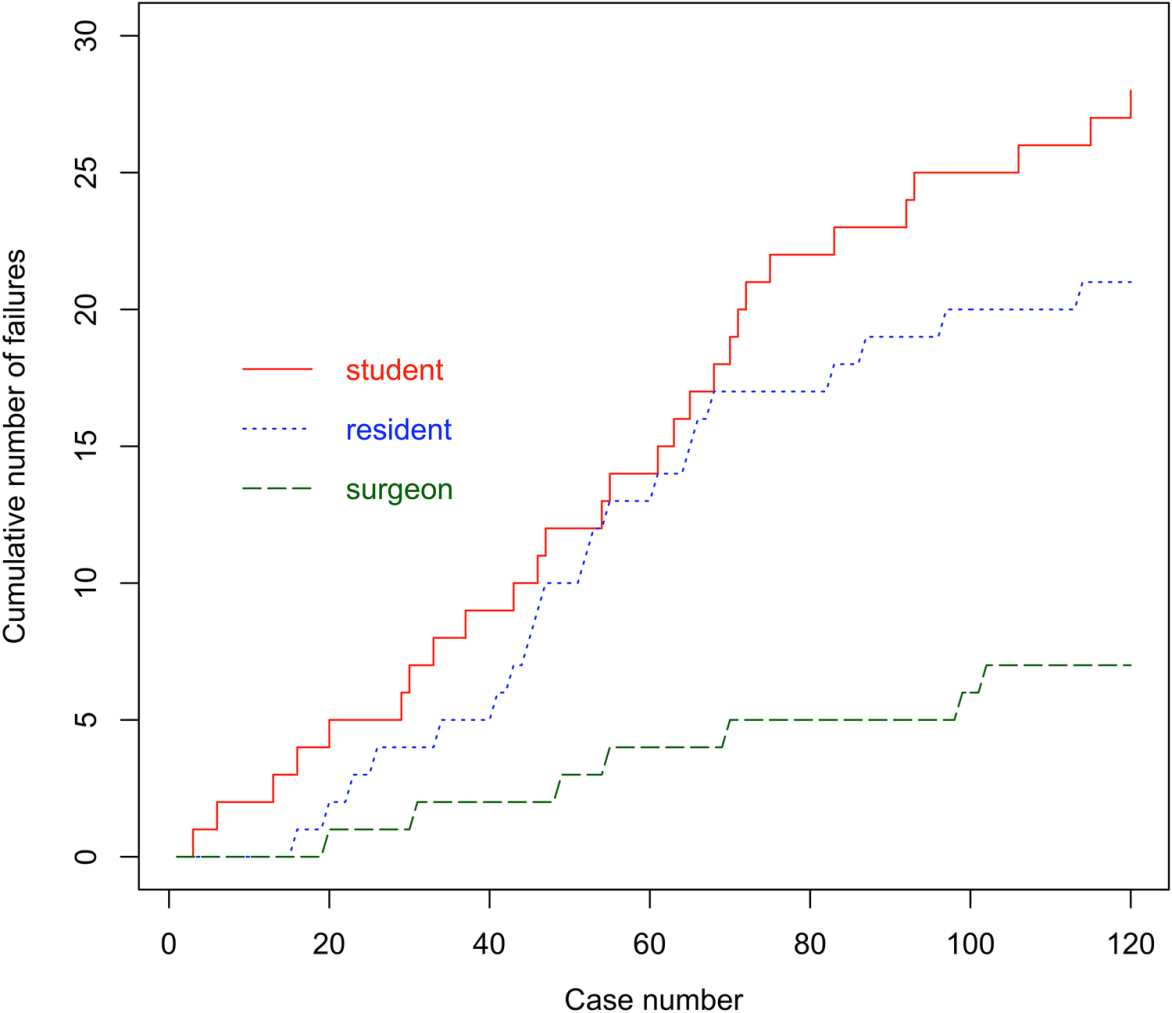
The cumulative number of failures of trainee 1 (student) and trainee 2 (resident) with reference to the definitely used cup size

With reference to the definitely used cup size, trainee 1 had 12 outliers in his early 50 attempts, and 13 outliers in his late 50 attempts, respectively. Trainee 2 had 10 outliers in his early 50 attempts, as well as 10 outliers in his late 50 attempts. The cumulative number of failures of the surgeon was low with 3 outliers in his early 50 cases and 3 outliers in his late 50 cases, respectively (Fig. 3).

### LC-CUSUM/CUSUM

**Fig. 4:**
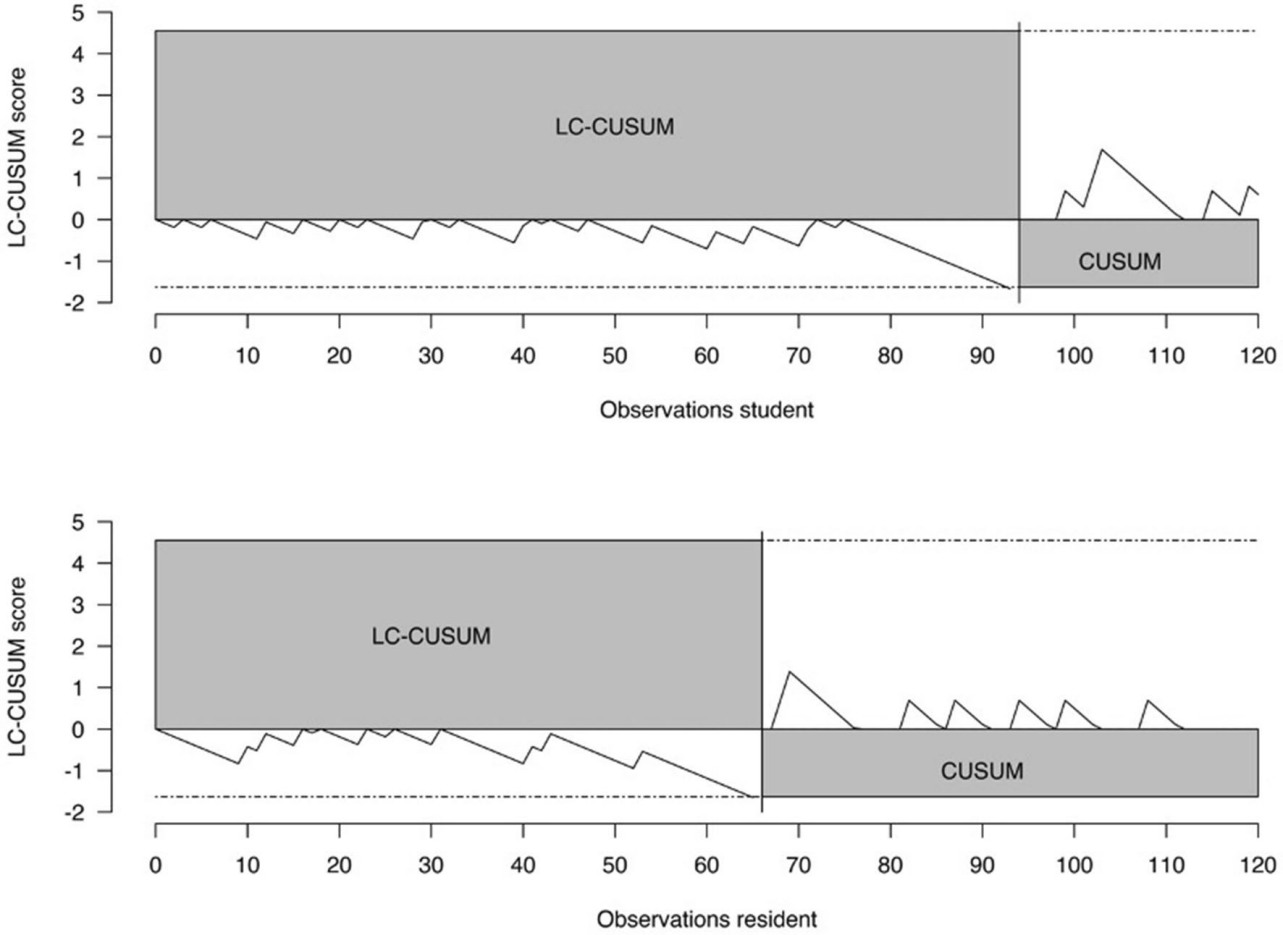
The LC-CUSUM test signaled competency after 66 attempts for trainee 2 (resident, bottom graph) and after 94 attempts for trainee 1 (student, top graph), respectively. The resident and the student reached competency within the first planning attempts. Both trainees retained competency thereafter

**Fig. 5:**
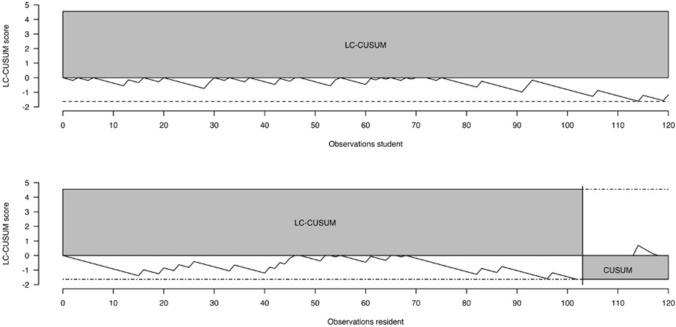
The LC-CUSUM test did not signal competency within the first 120 attempts for trainee 1 (student). The LC-CUSUM test signaled competency after 103 attempts for trainee 2 (resident); he retained competency thereafter

With reference to the preoperative plan of the surgeon, LC-CUSUM indicated proficiency after 94 planning attempts for trainee 1 and proficiency after 66 attempts for trainee 2, respectively (Fig. 4). Trainee 1 and 2 maintained proficiency thereafter. With reference to the definitive cup size, LC-CUSUM did not signal competency within the first 120 planning attempts for trainee 1. Trainee 2 was declared competent after 103 attempts and he retained competency thereafter (Fig. 5).

## Discussion

The importance of preoperative planning in total hip arthroplasty is widely accepted [8, 11, 21]. The restoration of physiological joint biomechanics in contemporary THA requires a detailed preoperative assessment of the bony morphology, the arthritic wear pattern, and an evaluation of the appropriate implant design and size [10, 12]. Preoperative planning may also help to reduce the number of intraoperative complications such as periprosthetic fractures [11, 21]. Preoperative planning may help to avoid excessive medialization of the cup which is associated with inferior outcomes following THA [5]. Accurate templating gains additional relevance when no intraoperative radiographs are used for primary cementless THA. In the present study, AP pelvis radiographs were used because they allow for an accurate assessment of acetabular offset [15].

In the clinical practice of preoperative templating, the prediction of the definite implant size ± one size is considered acceptable [8]. Analog and digital films may be used for preoperative planning with comparable accuracy [11, 25]. Double markers did not increase the accuracy of image calibration [27]. According to the literature, the definite acetabular cup size is predicted within ± one size in 80%–100% [8, 11, 21, 24, 26]. However, these calculations are unsatisfactory because they only allow for conclusions of whether a predefined goal was reached in the end. Variations in the performance during the time period under examination—improvement or deterioration in planning accuracy- are not detected using these simplistic calculations. Advanced methods such as LC-CUSUM and the CUSUM test are necessary to allow for an individualized, quantitative and continuous assessment of templating quality and the related learning curve [2, 3]. To the best of our knowledge, this is the first study that applied LC-CUSUM to evaluate the learning curve associated with a non-manual (i.e. surgical) task.

The present study shows that previous experience positively influenced the performance of preoperative templating. The least experienced trainee 1 (student) achieved an acceptable (± one size) templating rate of 77% (92/120). Therefore, the predefined performance rate of 85% was not reached taking the entire cohort of 120 patients as calculation base. However, LC-CUSUM indicated that the student assessed in this study attained proficiency after 94 planning attempts with reference to preoperative templating of the senior surgeon. The CUSUM test showed that attained proficiency was maintained thereafter. LC-CUSUM/CUSUM, therefore, allowed for a more accurate and individualized assessment of the learning curve during the period of observation, and a continuous assessment of the performance once proficiency was attained. With reference to the definite cup size, LC-CUSUM did not signal competency during the first 120 planning attempts for the student. In the present study, the preoperative radiographs with the cup template were visible after the size was determined. The postoperative radiographs were not available. The fact that student could be declared competent with reference to the preoperative template but not competent with reference to the definite implant size shows that a continuous feedback (visual assessment of the surgeons’ templating) positively influences the learning process.

Trainee 2 was a senior orthopedic resident with previous experience in THA templating and surgery. Despite the experience, the overall performance rate was only 83% and did not reach the desired accuracy of at least 85%. However, analyzing the learning curve, LC-CUSUM indicated proficiency in the planning of the acetabular cup size after 66 attempts with reference to the preoperative templating of the senior surgeon. Subsequently, attained proficiency was maintained. With reference to the intraoperatively used cup sizes, trainee 2 reached and maintained proficiency after 103 attempts. An explanation for the high failure rate up to attempt 103 is that trainee 2 had previously learned a more ‘anatomic’ implantation technique where reaming is performed to get just enough circumferential bony cup coverage. In the current study, however, the ‘conventional’ medializing reaming technique served as benchmark. Therefore, the resident had to adopt his previous planning technique and ‘learn’ a different technique. Interestingly, LC-CUSUM/CUSUM was able quantify this dynamic learning process.

The present study is the first to quantify the individual learning process of determining the acetabular cup size in preoperative templating. The study is clinically relevant because it shows that LC-CUSUM can successfully be applied to assess the learning process of non-surgical tasks. Furthermore, adaptations of LC-CUSUM/CUSUM would allow to immediately identify factors such as BMI, lumbar spinal fusion or hip contractures that may influence the accuracy of preoperative planning. The failure rates with reference to the definite implant size were similar for women and men.

### Limitations

(1) The current study did not evaluate the learning curve of preoperative templating of the femoral component. (2) Individual factors (e.g. BMI, gender or pelvic tilt) influencing the accuracy of the preoperative plan were not taken into consideration. (3) The individual learning process of only two trainees was assessed. Analyzing more trainees may allow for a generalization on how many planning attempts are necessary for residents to become competent. However, this was beyond the scope of the present study.

## Conclusions

LC-CUSUM/CUSUM allow for an individualized, quantitative and continuous assessment of templating quality. Based on LC-CUSUM statistics, the two trainees of this study gain proficiency in planning of the acetabular cup size after 50–100 attempts when a concise algorithm and an immediate feedback is provided. Previous experience positively influences the performance. The study serves as basis for the medical education of students and residents in joint replacement procedures**.** LC-CUSUM/CUSUM are a powerful tool the enhance the preoperative planning quality to minimize the intraoperative risk leading to a successful outcome for the patient.

## References

[1] Akbar M, Aldinger G, Krahmer K, Bruckner T, Aldinger PR (2009). Custom stems for femoral deformity in patients less than 40 years of age: 70 hips followed for an average of 14 years. Acta Orthop.

[2] Biau DJ, Porcher R (2010). A method for monitoring a process from an out of control to an in control state: application to the learning curve. Stat Med.

[3] Biau DJ, Williams SM, Schlup MM, Nizard RS, Porcher R (2008). Quantitative and individualized assessment of the learning curve using LC-CUSUM. Br J Surg.

[4] Campbell RD, Hecker KG, Biau DJ, Pang DS (2014). Student attainment of proficiency in a clinical skill: the assessment of individual learning curves. PLoS ONE.

[5] Cech A, Kase M, Kobayashi H (2020). Pre-operative planning in THA. Part III: do implant size prediction and offset restoration influence functional outcomes after THA?. Arch Orthop Trauma Surg.

[6] Dagash H, Chowdhury M, Pierro A (2003). When can I be proficient in laparoscopic surgery? A systematic review of the evidence. J Pediatr Surg.

[7] de Oliveira Filho GR (2002). The construction of learning curves for basic skills in anesthetic procedures: an application for the cumulative sum method. Anesth Analg.

[8] Gonzalez Della Valle A, Comba F, Taveras N, Salvati EA (2008). The utility and precision of analogue and digital preoperative planning for total hip arthroplasty. Int Orthop.

[9] Hasegawa K, Kabata T, Kajino Y, Inoue D, Tsuchiya H (2017). Periprosthetic occult fractures of the acetabulum occur frequently during primary THA. Clin Orthop Relat Res.

[10] Kase M, O'Loughlin PF, Aït-Si-Selmi T, et al. (2020) Pre-operative templating in THA. Part I: a classification of architectural hip deformities. Arch Orthop Trauma Surg, 140(1):129–137 10.1007/s00402-019-03298-1 31696320

[11] Knight JL, Atwater RD (1992). Preoperative planning for total hip arthroplasty. Quantitating its utility and precision. J Arthroplasty.

[12] Kobayashi H, Cech A, Kase M (2020). Pre-operative templating in THA. Part II: a CT-based strategy to correct architectural hip deformities. Arch Orthop Trauma Surg.

[13] Lewinnek GE, Lewis JL, Tarr R, Compere CL, Zimmerman JR (1978). Dislocations after total hip-replacement arthroplasties. J Bone Joint Surg Am.

[14] Meermans G, Malik A, Witt J, Haddad F (2011). Preoperative radiographic assessment of limb-length discrepancy in total hip arthroplasty. Clin Orthop Relat Res.

[15] Merle C, Innmann MM, Waldstein W (2019). High variability of acetabular offset in primary hip osteoarthritis influences acetabular reaming-a computed tomography-based anatomic study. J Arthroplasty.

[16] Merle C, Waldstein W, Pegg E (2012). Femoral offset is underestimated on anteroposterior radiographs of the pelvis but accurately assessed on anteroposterior radiographs of the hip. J Bone Joint Surg Br.

[17] Merle C, Waldstein W, Pegg EC (2013). Prediction of three-dimensional femoral offset from AP pelvis radiographs in primary hip osteoarthritis. Eur J Radiol.

[18] Miettinen SS, Makinen TJ, Laaksonen I (2017). Early aseptic loosening of cementless monoblock acetabular components. Int Orthop.

[19] Page ES (1954) An Improvement to Wald's Approximation for Some Properties of Sequential Tests. Journal of the Royal Statistical Society, Series B

[20] Ramsay CR, Grant AM, Wallace SA, Garthwaite PH, Monk AF, Russell IT (2000). Assessment of the learning curve in health technologies. A systematic review. Int J Technol Assess Health Care.

[21] Shaarani SR, McHugh G, Collins DA (2013). Accuracy of digital preoperative templating in 100 consecutive uncemented total hip arthroplasties: a single surgeon series. J Arthroplasty.

[22] Sharkey PF, Hozack WJ, Callaghan JJ (1999). Acetabular fracture associated with cementless acetabular component insertion: a report of 13 cases. J Arthroplasty.

[23] Steinberg EL, Shasha N, Menahem A, Dekel S (2010). Preoperative planning of total hip replacement using the TraumaCad system. Arch Orthop Trauma Surg.

[24] Suh KT, Cheon SJ, Kim DW (2004). Comparison of preoperative templating with postoperative assessment in cementless total hip arthroplasty. Acta Orthop Scand.

[25] The B, Verdonschot N, van Horn JR, van Ooijen PM, Diercks RL (2007). Digital versus analogue preoperative planning of total hip arthroplasties: a randomized clinical trial of 210 total hip arthroplasties. J Arthroplasty.

[26] Unnanuntana A, Wagner D, Goodman SB (2009). The accuracy of preoperative templating in cementless total hip arthroplasty. J Arthroplasty.

[27] Warschawski Y, Shichman I, Morgan S (2020). The accuracy of external calibration markers in digital templating using the double marker and single marker method: a comparative study. Arch Orthop Trauma Surg.

